# Analysis of the Exploration of Security and Privacy for Healthcare Management Using Artificial Intelligence: Saudi Hospitals

**DOI:** 10.1155/2022/4048197

**Published:** 2022-09-14

**Authors:** Abdulmohsen Almalawi, Asif Irshad Khan, Fawaz Alsolami, Yoosef B. Abushark, Ahmed S. Alfakeeh, Walelign Dinku Mekuriyaw

**Affiliations:** ^1^Computer Science Department, Faculty of Computing and Information Technology, King Abdulaziz University, Jeddah 21589, Saudi Arabia; ^2^Department of Information Systems, Faculty of Computing and Information Technology, King Abdulaziz University, Jeddah 21589, Saudi Arabia; ^3^Department of Chemical Engineering, College of Biological and Chemical Engineering, Addis Ababa, Ethiopia

## Abstract

A large component of the Health Information Systems now comprises numerous independent apps created in the past that need to be merged to provide a more uniform service. In addition to affecting the Intelligent Health Board Functionality and dependability, the quality of these additional apps may also have an impact. A critical characteristic of the SHS's management and upkeep is the SHS's reliance on the real benefits provided to it. In speaking, an HMIS (Healthcare Management Information System) is a computer-based device that benefits medical practitioners to perform their duties more efficiently by coordinating all of their data. Even though these systems are widely used by most of the world, there is a significant need to comprehend these technologies and indeed the potential they provide. Healthcare data warehouses in Saudi Arabia have evolved through time, and this research examines how key service improvements in Saudi present varied viewpoints on how premium initiative help may be attained in health as well as how this could be done. When it comes to understanding how different types of medical professionals interact with healthcare systems throughout history, researchers developed stages of the maturity model.

## 1. Introduction

Creating legally acceptable systems is a difficult software engineering task, particularly for systems that are subject to regulatory restrictions, like healthcare information systems. The ambiguity and domain-specific definitions present in governmental regulations are the source of this difficulty. Because of this, there is a critical business need to automatically assess privacy texts, extract rules, and then apply those rules throughout the supply chain. The US Health Insurance Portability and Accountability Act is used as an example in the current works that analyze health policies. Saudi Arabia's healthcare data warehouses have developed over the years, and this study looks at how important service enhancements in Saudi offer different perspectives on how premium initiative help in health may be accomplished as well as how this may be done. Researchers have created stages of development model to help explain how various medical practitioners have interacted with healthcare systems throughout history.

Patient medical and nursing administration are the primary functions of hospitalization strategic planning (HMIS). In addition to maintaining track of patients and their medical histories, the hospital also maintains a record of vaccinations prescribed to treat, as well as knowledge of different illnesses and the medications that may be used to treat them [1]. All of these many tasks were formerly handled by hand by the operational cadre and physicians. As the demand for healthcare resources developed as a result of an increasing economy and an increased focus on individual protection and treatment approaches, it became more challenging to consistently complete all of these obligations. In addition to rapid and varied improvements in ICT, which hold a position of leadership and are a vital component in the shift from a traditional to a digital system, electronic health information management has become a necessity to automate all of the other operations. Failure to comply with these regulations might have serious financial and reputational repercussions for healthcare providers in addition to the risk of sensitive patient information being disclosed, which could harm patients' reputations and finances. Because of this, creating a legally compliant system is a difficult software engineering task and is acknowledged as a major problem, especially in systems governed by law. Ambiguities and domain-specific definitions present in governmental regulations are the source of this problem. For instance, Saudi legislators intended for ambiguities in healthcare privacy legislation to be reinterpreted as business practices change and people's capacity to comply with regulations changes over time.

The promise of artificial intelligence (AI) to improve certain facets of healthcare is already being fulfilled. It will probably become crucial in the future to support clinical and other applications that lead to more intelligent and efficient operations and care. With more secure and interoperable data, AI is going to be a key driver of analytics, insights, and the decision-making process across health plan, pharmacy benefit manager (PBM), and health system companies today. Businesses that embrace adoption are likely to see immediate benefits from cost savings and long-term competitive advantage as a result of using AI to alter their goods and services and improve customer engagement.

It was characterized as a comprehensive personal computer for the storage, manipulation, management, and retrieval of medical and administrative health data organizations [2]. To better understand a healthcare facility's IT infrastructure, consider it to be more than simply machines, Internet, and machine infrastructure components. It is about so many doctors' data and its ability to handle and store it. When it comes to medicine, it has the potential to cut expenses and enhance performance while eliminating the problems of inaccuracies in data storage, reporting, and retrieval that conventional facilities face. Benefits aside, the journey to implementing this ideal system is not without difficulties [3]. Some of these issues stem almost from the character of patient data, while others stem from complicated health informatics and its users. Hospital software has been the subject of several studies by academicians and practitioners from a wide range of fields.

When it comes to running a facility's operational, budgetary, and surgical operations, a HIS is an indispensable tool [4]. By using computerized data processors to enhance clinical outcomes and administrative processes, healthcare information systems are a subset of health imaging [5]. The healthcare industry has benefited greatly from the use of information technology. Computational modeling and accompanying applications have been introduced into the medical environment throughout the early 1990s. Organizations are spending a lot of money on online filing capacities and kinds of community hospitals to remove costly, bulky, and challenging paper health history with digital information that will be much faster and more efficient and integrate numerous duties.

As a result, the health information system (HMS) plays a very important role in designing, implementing, organizing, and overseeing the processes of the doctor's subdomains and so enables constructive goals and business objectives of implementing the hospital's strategic plan and achieving its goals [6]. Using HMS, hospitals may transition from one historical to a continuous evaluation and improvement and proportionality of treatment, which increases the nursing experience. HMS has been implemented at a large number of Saudi hospitals, spread out over many cities in the region. In Saudi Arabia, the Ministry of Health (MOH) is the most prominent government agency and the principal provider of medical services. About 98 percent of all health expenses are funded by the MOH. Health information Systems (HIS) have been given high attention in Saudi healthcare institutions because of the growing consciousness of the need for improved treatment and also the consequent growing adoption.

Many nations, including Saudi Arabia, are seeing an increase in sales for pharmaceutical tests and procedures due to reasons along with an aging population and the increasing citizen expectations for good health and well-being [7]. This is the case in Saudi Arabia as well. The subject of healthcare has become more important to various governments. To ensure that all residents have access to an affordable, high-quality healthcare system, government and international healthcare systems have been implemented throughout the last several decades. For regional medical professionals, these policies have presented a thorny issue. As a result of a variety of changes happening during the last two decades, the administration of hospitals has to adapt their computer networks. The main difference is that, by emphasising significant service innovations in Saudi Arabia, this suggested work offers many viewpoints on how cost-effective service excellence in healthcare may be attained. As a result, fresh lines of inquiry have been discovered. Healthcare Method is conducting this research to create a smart and safe system for managing medical records. This system should facilitate the entry of patient data, improve the management of doctor appointments, facilitate the flow of information across departments, and accurately archive patient data and diagnoses.

## 2. Objective

The main purpose of this article is that key service innovations in Saudi Arabia give varied viewpoints on how cost-effective service excellence in healthcare might be accomplished. As a result, new research avenues have been identified. Healthcare System is conducting this study to create a smart and protective system for managing healthcare records. This system should make it easier to create patient data, better manage doctor schedules, allow information to flow smoothly from one department to the next, and precisely archive patient information and diagnoses.

## 3. Review of Literature

Marzouk and Othman [1] showed that institutional arrangements may be necessary for social sustainability to ensure that establishments are responsive to future requirements. Effects minimization was first used in previous research as a way to evaluate institutional practice. It is up to HCIS administrators such as clinics and local medical organizations to guide the road toward sustained growth by ensuring equitable care and preventing unneeded medication, which will be operating efficiencies to decrease the environmental effect. Analysis from Italy reveals that it is essential to enable helping the transition of health doctors to the green economy and stimulate deeper exploration of HCIS's planning process for durability.

In [2] Medical Healthcare Initiative1 also includes services and resources for the future. Environmental accounting is provided by the World Development Initiative2 to assist firms quantify, analyze, and explain their impact on critical sustainable solutions. Companies may use the Dollar Index Correction Factor to gauge the health of their financial position as a part of the recent opportunities and risks associated with decarbonization. Many other indications and evaluation techniques may be used for attention and cognitive HCIS.

In addition to [3], to better comprehend the outcome measures of healthcare data (HIS), [8] focuses on synthesizing the newest generally recognized systems and procedures including Healthcare Level 7 (HL7) specifications for simultaneous packet forwarding, HIS subsystems, etc. As a result, much of the data included in the research comes from reliable information of data collected. E-Hospital Leadership and even the doctor's office Management System include several parameters, and now one of these was recognized by academics as Disaster Response.

Kshirsagar et al. [4] improved identification and model accuracy by combining hybrid machine learning with Pareto-optimal solutions for a wide variety of information, such as standard performance and feature sets from a variety of growing domains [9, 10]. The methodologies employed in numerous research projects were beneficial yet more again among diverse assessment criteria in information technology, computational science, and cloud-based services. In [9] *K*-means segmentation method primarily sought to examine Delhi's polluted air and determine the source of the substances that may pollute the atmosphere. Ashok Vihar, R.K. Puramand, and Punjabi Bagh are of the most contaminated areas, according to the researcher.

Moreover, in [5] half of China's emergency rooms are county institutions, according to government figures from 2018 of one of four hospitals in the county-level public health system in western China, the state hospital would be the best level of treatment available. Postsecondary care is available in most circumstances at these facilities. Pharmaceutical care is given to approximately 900 million people in other countries, or maybe more than 70 percent of the global total, via county institutions. Healthcare bills at county facilities began in 2012 to increase inpatient beds for improved rural medical services by enhancing community healthcare finance, effectiveness, and convenience in service provision and care managers. In 2014, the transformation was extended to over 1,000 county institutions, and in 2015, it was extended to every medical center. We needed to create a contemporary patient care system to help us better manage our facilities. To do this, we needed to enhance our governance, streamline our business, develop an accurate quality assurance system, and raise the wages of our employees.

## 4. Improvements in Healthcare Management

### 4.1. National Healthcare

In Saudi Arabia, the Ministry of Health is the primary government department concerned with the administration, planning, funding, and regulation of the healthcare industry. In addition to this, it is responsible for the overarching monitoring and follow-up of all activities linked to healthcare that are carried out by the private sector. The Saudi healthcare system is ranked 26th among 190 of the world's health systems according to the World Health Organization (WHO). Saudi Arabia's healthcare system includes medical services, hospital treatment, and medical specialist treatment. At the present time, the Ministry of Health in Saudi Arabia is the most important government provider and financier of healthcare services in the country. With a total of 244 hospitals containing (33277 beds) and 2037 primary healthcare clinics, the MOH is responsible for the healthcare infrastructure in Saudi Arabia (PHC). The MOH oversees twenty regional directorates-general of health affairs across the nation. Each regional health directorate supervises a number of hospitals and health sectors, and each health sector is responsible for a number of PHC centers. These 20 directorates are responsible for executing the policies, plans, and programmes of the Ministry of Health, administering and supporting MOH health services, monitoring and organizing private sector services, and collaborating with other government agencies and relevant authorities [8, 11]. As a result, organizations have indeed been assigned a predetermined budget for the facilities and services they provide regularly.

### 4.2. The Hospital's Most Important Functions

**Figure 1:**
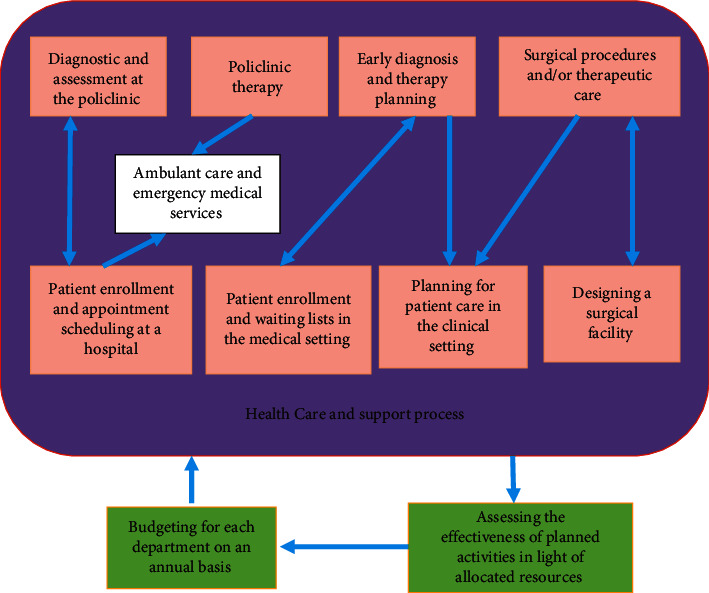
Relationships between the two mechanisms in health facilities.

The lack of indigenous healthcare professionals, such as doctors, nurses, and pharmacists, is a challenge for the Saudi healthcare system. It is of the utmost importance to devise and put into action workable strategies and protocols in order to satisfy the requirements posed by both patients and those employed in the healthcare industry. Because most of the people working in the healthcare industry are foreign nationals, there is a significant employee turnover rate, which contributes to an unstable workforce. Policy-makers and researchers in Saudi Arabia agree that the most effective option to revamp the country's healthcare system would be to privatize the country's public hospitals [11, 12].  Figure 1 illustrates how the specialized healing method and the treatment and protection method, which are the main two things that are happening in hospitals, are related. The estimate shows that two key steps share information about patients and the healthcare workers themselves. This makes it hard to make sure the diagnosis, care, and assistance are all of the excellent quality, and it also makes it hard to exchange intelligence with planning [9]. These problems are made worse by the fact that doctors run their practices like private businesses in hospitals.

### 4.3. Hospital Management

Hospitals in Saudi Arabia are in charge of their administration within the limits of national laws and rules. Healthcare development for the short and longer term is influenced by state and regional developments, and medical appropriations are depending upon which options are available. For planning diagnostic and outdoor services, hospitals talk with the local government and insurance companies [13]. Healthcare management is a key “middleman” in better healthcare networks in the United States. Nationally, the law passed very precise budget cutbacks, restricts the frequency of some treatment recommendations, and requires individuals to pay for these kinds of treatments with their wallets in addition to keeping medical costs down.

Rather than these series of strategies, healthcare facilities prefer more global and long-term measures from the administration, so they can plan their hospitals better for national and environmental protection laws. Hospitals are like specialist bureaucrats. Medical facilities can also be seen as organizations that depend heavily on technologies and knowledge. Lawrence and Dyer say that all these sorts of groups are not government departments that are set up in a hierarchy [14]. Rather, they are often based on representative democracy regulatory systems that give people who have a shareholding in the judgment procedure a formal manner to have their say. It would be fun to discover if medical facilities are run by bureaucracies or by individuals. We have come up with three types of healthcare managers to assist us with this inquiry. These allow development, functional management, and network monitoring.  Table 1 shows identification of three types of hospital management.

## 5. Classifications of Smart Healthcare

Healthcare systems in place today simply cannot keep up with the ever-increasing demand. Medical services are not accessible or cheap to everyone, even though the framework and slashing technology are in place to support them. A primary objective of smart healthcare is to keep patients informed and involved in their care by providing them with timely and accurate information about their medical conditions and treatments [15]. Many critical scenarios may be handled by the user in an intelligent healthcare system. Users' quality and satisfaction are prioritized in this approach. To make the most use of the resources that are already in place, smart healthcare is necessary. It facilitates remote patient monitoring and reduces the cost of therapy for the user. The ability to provide care to patients across national borders is made possible by this technology [16]. A city's residents must have access to quality healthcare to maintain a healthy lifestyle in today's increasingly intelligent cities.

**Figure 2:**
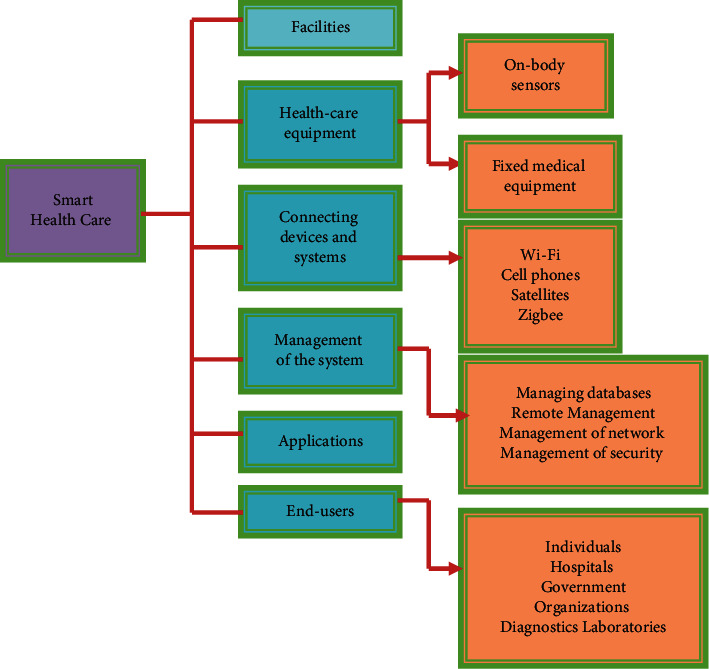
Classification of smart healthcare.

Customer satisfaction is done with the offerings they get as well as the systems they use to access them.  Figure 2 depicts how the smart healthcare industry may be divided up into many categories. Technologies of connectivity are critical in broadening the scope of healthcare's intended uses. Using wireless technology, tiny devices may be integrated into the Internet of Things to provide remote health monitoring [17]. A Bluetooth, 6LowPAN, or RFID chip may be used to link a wristband style personal monitoring gadget to the Internet for remote viewing. For example, at a healthcare facility, connectivity through Wi-Fi and underground cables are essential for sustaining high data flow [18]. On-body scanners and immobile diagnostic implants seem to be the two most common types of smart medicine diagnostic instruments in relation to medical electronics. For monitoring patients, biosensors connected to the body are sometimes referred to as “on-body sensors.”

### 5.1. Attributes of Smart Healthcare

**Figure 3:**
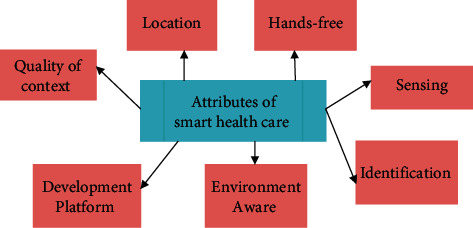
Attributes of smart healthcare.

The components and settings that the medical infrastructure is utilizing make up a framework for smart healthcare architecture. Network platforms, computer systems, and application servers are all types of medical portals. Interconnecting multiple designs is done via the usage of network platforms. A computer's operating system might be very different depending on the technology employed [19]. When it comes to medical surveillance, there are many different program settings and therefore a wide range of smartphones and computers that may be used to implement the various components of a real-time application. The middleware layer serves as an intermediary between technology and the end user. If you are using a more sophisticated application, you may use robots or algorithms with a behavioral and cognitive viewpoint to provide this support layer [20]. The Internet of Things (IoT) may be used to handle health data. Before modeling systems, organizations, and platforms, it is important to examine the following qualities.  Figure 3 shows attributes of smart healthcare.

## 6. Methodology

**Figure 4:**
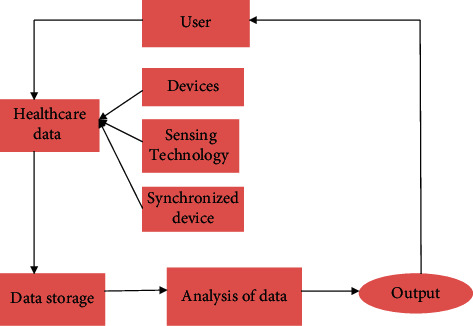
Healthcare system flowchart.

The primary goal of this system is to give an interactive environment for its user and some improved medical facilities with big data analysis and to enhance medical healthcare quality.  Figure 4 depicts the operation's flow diagram, which focuses primarily on the data-collecting strategy to allow for human-machine interactions. The body's important information is kept in medical data records at the health centers depending on the patient's medical history. In this method, the doctors or other responsible medical staff will continuously monitor their patients' data, inform them to visit a health center immediately if necessary, and take other specific actions, such as using telemedicine services, as necessary. Additionally, if the situation is urgent or very critical, the health centers can contact the hospitals to provide rapid medical services, such as ambulance service. In the suggested system, we will collect and analyze the biological data of our users in order to improve healthcare. Because of this, the most difficult component of the new healthcare system is the study and collection of the most important information from the vast biological raw data [21]. Human-computer contact is vital for the system to get more biological data, allowing it to explore more relevant information. To diagnose an illness, the new healthcare system uses an analysis of the link between a patient's biological state, mood, and evolving health status, as well as past health information, to improve interactions between doctors and patients. A novel interactive system with big data analysis in the healthcare system has been suggested in this part. The output of the system and the data storage and analysis stored in the cloud complete the system's functionality.

The data will be transmitted to the cloud for analysis and processing once it is collected and stored locally. There, we will look for patterns in the data using MLA and DM techniques. Machine learning algorithms may forecast illness based on data from specific test cases. In the cloud, unstructured data may be available. A more advanced algorithmic foundation is required. Using the anticipated method, the expected outcome will be compared to past health data and family health data [22]. An additional report is also generated by the system. In this report, we can see how our health has changed over time. All output will be transmitted to the user's mobile device or personal PC [23]. This approach, which measures probabilistic categorization, makes it simpler to make a diagnosis. Based on the patient's medical history, predicted diseases are employed in conjunction with this technique. We will utilize this approach once again to get information about the condition of our family members [24–33].

### 6.1. Data Collection

For something like the purposes of calculating the HMP value, clinical service manager's questionnaires were administered. A neurosurgeon and a president of pharmaceutical and biotech leadership at each cooperating institution were requested to fill out questions created by them to acquire data about their divisions [34]. The lengthy vision, annual improvement plan, effective assessment plan, and patient experience monitoring legislation have all been obtained throughout our investigation. The surveys were delivered and received in separate, sealed envelopes to preserve the integrity of the data. Researchers and hospital administrators alike received the same education.

### 6.2. Measuring Healthcare Outcomes, Effectiveness, and Cost

Each hospital's medical affairs management office completed a questionnaire to gather information about the quality of treatment, efficiency, and finances. A hospital's mortality rate and the incidence of hospital-acquired infections serve as quality-of-care measures. To measure efficiency, we looked at how long a patient had to remain, how many beds were available, and how many patients were in each bed at any one moment [10, 35]. Costs per hospitalization and yearly total revenue for a hospital were two financial indicators. The yearly data of the hospital is reflected in all measures [36].

### 6.3. Covariates

Several factors might affect a clinic's ability to care for its patients, including its location, its academic capacity, its competition, or the number of workers and spaces that are on hand [7]. Competitors were defined as other medical facilities in the same county that provide services at the same technical level as the hospital in question [35]. “Staff” may refer to any member of the hospital's workforce, including medical professionals as well as office workers and other support personnel [37].

## 7. Result and Discussion

In the initial survey, 85 of the 100 eligible hospitals participated; in the subsequent study, 93 of the 100 hospitals did so. In total, 96 hospitals took part in the study, and each of them completed at least one survey.  Table 2 lists the main characteristics of the participating medical facilities and uses statistical analysis to get the *p* value.

**Figure 5:**
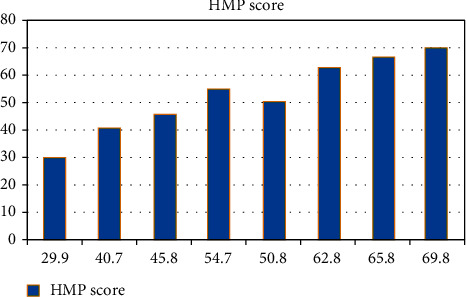
Distribution of HMP Score. The aggregate HMP score varied from 69.8 at the greatest to 29.8 at the lowest among the 96 participating institutions.

Figure 5 depicts the HMP distribution. The use of hypothermic machine perfusion (HMP) in cardiac death kidney transplantation is increasing, but it is still debatable whether HMP characteristics are useful for determining organ quality and anticipating delayed graft function (DGF). As a result, we created a scoring model using a widely available HMP variable that can identify those at the greatest risk of DGF and offer direction and recommendations for organ allocation and DCD kidney assessment.

It was determined that the combined HMP score ranged from 49.3 to 69.8 and that 83.8% of the 96 participating hospitals had an HMP score of less than 65 out of 100. The average score for each of the four elements of effectiveness and talent planning, which make up the four pillars of goal setting, was 378.

HMP score is evaluated using statistical tools. The formulas used are basic probability theorems like Bayes theorem.

Figure 5 and Table 3 demonstrate the distribution of HMP scores.

**Figure 6:**
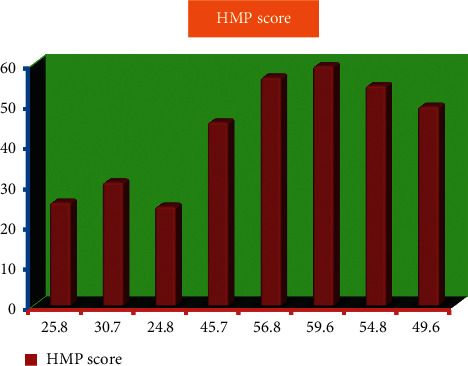
Distribution of HMP score.

Figure 6 and Table 4 demonstrate the distribution of HMP scores. The mean overall HMP score across 92 participating hospitals was 47.3 and varied from 59.6 at the highest to 24.8 at the lowest, according to the results.

### 7.1. Every Measurement and Indication, the Mean HMP Score from 96 Participating Hospitals

**Figure 7:**
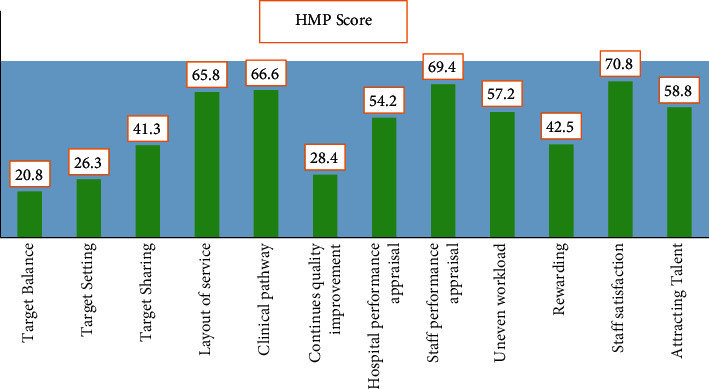
Mean HMP score for each dimension and indicator among 96 hospitals.

Figure 7 and Table 5 show the mean HMP scores per component and variable across 96 hospitals. In every one of the four functions of management, including specific populations, production management, quality management, and talent planning, each had an average rating of 20.8, 26.3, and 41.3, respectively.

Consequently, creating a legally compliant system is a difficult software engineering issue and is acknowledged as a major concern, particularly in systems governed by law. This difficulty is brought on by the vagueness and industry-specific terminology present in governmental regulations. For instance, Saudi politicians intentionally included gaps in its healthcare privacy laws so that they might be reinterpreted if business practices changed and people's capacity to follow the law changed. According to Regulation IV-3-1, for instance, “the physician may reveal some of the patient's secrets if they are necessary for educating physicians or other members of the medical team if it is necessary to do so”; the word “necessary” is purposefully ambiguous, as it is unclear precisely which secrets are considered necessary. Software engineers cannot eliminate this ambiguity, which stems from English terms that have several logical interpretations, from the legal requirements; rather, it can only be categorized and interpreted in the context of organizational processes, goods, and services (7). As a result, there is a substantial commercial requirement for tools that can automatically assess privacy texts, extract rules, and then apply those policies throughout the supply chain.

## 8. Conclusion

The primary objective of this study is to determine whether or not it will be possible to make use of the power of artificial intelligence in the analysis and exploration of security and privacy service in order to develop a more cost-effective healthcare system. For this data was gathered from all centers across the nation, a purposefully nonrandom distribution was adopted. Each of the provincial municipal treatment centers that was attended has its own distinctive characteristics. As a result, the study was carried out. Findings must be interpreted cautiously when applied to all Saudi hospitals. Also possible to be the result of a small sample are the insignificant connections. There will be more investigation in the future. It is essential if we are to be able to confirm our findings. Last but not least, we thoroughly investigated the business and its personnel. Concentrate the attention on the field of cardiologists; it is possible that other divisions are not doing as well as required. In our HMP assessment of a single doctor and three different management positions, we included a question on HMP to make the HMP assessment simple and practical. There were not any other types of workers mentioned, including nurses. There might be a trade-off even though future study might encompass other functions. Additionally, excessive labor increases must be acknowledged. Despite significant heterogeneity, according to the findings of our study, senior management of the quality of care provided at hospitals located in Saudi Arabia frequently exhibited low performance. The pluses and minuses in their patient care were the same for both the highest and lowest HMP ratings. The fundamental similarity between vulnerability and best practices in leadership is that there were no indications that things were going to get better. Hospitalization rates could be projected annually based on the quality of service and the total revenue sharing capacity. In order for Saudi Arabia's modern healthcare bill to be effective, hospital administration must adopt the following strategies. The processing of disputes where the conflict's cause is implicit in a series of occurrences will demand more advanced methodologies in the future.

## Figures and Tables

**Table 1:** Identification of three types of hospital management.

	Management of potential	Management that serves a purpose	Process management
Consumers 'expectations	Professionals from various fields	Subspecialists	A group of interconnected experts
Important elements for achieving success	Compatibility as well as security	Correct diagnosis and treatment	Happiness and contentment
Controllers of information	Medical specialists	Subspecialists in the field of medicine	Networks of medical professionals
Resources for information	Expertise and training in the medical field	Scientific process, excellent education, and scholarly articles	Digital and knowledge-based networks
Strategies for coordination	Financial tracking and monitoring	Management with a purpose	Interaction and standardization

**Table 2:** The location details of the participating hospitals.

Hospital characteristics	Total (*N* = 96)	Low HMP hospitals	High HMP hospitals	*p* value
East	33.8	24.9	43.8	0.024
Central	44.3	42.7	43.7	
West	20.4	30.5	11.7	

**Table 3:** Distribution of HMP score (*N* = 96).

S.No	Frequency	HMP score
1	5	29.9
2	10	40.7
3	15	45.8
4	20	54.7
5	25	50.8
6	30	62.8
7	35	65.8
8	40	69.8

**Table 4:** Distribution of HMP score (*N* = 92).

S.No	Frequency	HMP score
1	5	25.8
2	10	30.7
3	15	24.8
4	20	45.7
5	25	56.8
6	30	59.6
7	35	54.8
8	40	49.6

**Table 5:** Mean to every measurement and indication, the mean HMP score from 96 participating hospitals.

S.No	HMP indicators	HMP score
1	Target balance	20.8
2	Target setting	26.3
3	Target sharing	41.3
4	Layout of services	65.8
5	Clinical pathway	66.6
6	Continues quality improvement	28.4
7	Hospital performance appraisal	54.2
8	Staff performance appraisal	69.4
9	Uneven workload	57.2
10	Rewarding	42.5
11	Staff satisfaction	70.8
12	Attracting talent	58.8

## Data Availability

The datasets used and/or analyzed during the current study are available from the corresponding author on reasonable request.
